# A case report on complete cure of recurrent primary canaliculitis by 4-snip punctoplasty and canalicular curettage

**DOI:** 10.1097/MD.0000000000013508

**Published:** 2018-12-10

**Authors:** Min Ho Kim, Ho Ra

**Affiliations:** Department of Ophthalmology and Visual Science, College of Medicine, The Catholic University of Korea, Seoul, Republic of Korea.

**Keywords:** canalicular curettage, primary canaliculitis, recurrent canaliculitis, 4-snip punctoplasty

## Abstract

**Rationale:**

For the treatment of primary canaliculitis, 1,2,3-snip punctoplasty and canalicular curettage are commonly used; however, a recurrence rate of 6.6% to 22% has been reported. Herein, we describe a case of recurrent primary canaliculitis that was completely cured by 4-snip punctoplasty and canalicular curettage.

**Patient concerns:**

A 53-year-old woman was admitted to our hospital with chief complaints of epiphora, discharge, eyelid flare up, and swelling near the inferior lacrimal punctum in the left eye, which initially presented 6 months earlier.

**Diagnosis:**

Based on the aforementioned symptoms, the patient was initially diagnosed with bacterial conjunctivitis at a local ophthalmologic clinic and used antibiotic eye drops for 6 months. However, her symptoms did not improve and they worsened at 2 weeks prior to admission. She was subsequently diagnosed with chronic dacryocystitis and referred to our hospital for surgical treatment. Slit lamp examination results showed conjunctival congestion in the inner corner of the left eye, along with eyelid flare up, swelling near the inferior lacrimal punctum, and yellowish discharge and concretion from the lacrimal punctal orifice. Furthermore, punctal regurgitation was not observed in the lacrimal sac compression test. Thus, the patient was diagnosed with primary canaliculitis on the basis of her clinical symptoms and laboratory findings.

**Interventions:**

Based on the diagnosis of primary canaliculitis, 1-snip punctoplasty and canalicular curettage were performed, and the patient was prescribed oral antibiotics for 2 weeks together with topical antibiotics for 4 weeks. Overall, the patient's symptoms improved after surgery, but epiphora and yellowish discharge from the lacrimal punctal orifice developed again 2 months after surgery during outpatient follow-up. Based on the diagnosis of recurrent primary canaliculitis, 4-snip punctoplasty and canalicular curettage were performed, and the patient was prescribed oral antibiotics for 2 weeks together with topical antibiotics for 4 weeks.

**Outcomes:**

Over a 6-month follow-up period, the symptoms disappeared completely and no other findings were observed.

**Lessons:**

Four-snip punctoplasty and canalicular curettage are simple clinical procedures that can minimize the recurrence rate of primary canaliculitis. Hence, 4-snip punctoplasty and canalicular curettage should be considered as the 1st-line treatment for primary canaliculitis and recurrent cases.

## 1 Introduction

Primary canaliculitis is a rare disease characterized by infection in the lacrimal canaliculus. It accounts for approximately 1.4% to 2% of all lacrimal diseases.^[1–3]^ Typically, it is more prevalent among women than among men, which may be due to reduced tear secretion in postmenopausal women who are more vulnerable to infections as a result of hormonal changes.^[1,3–7]^ Its clinical signs commonly overlap with those of other diseases that occur near the lacrimal apparatus; therefore, there are often cases in which proper treatment is not provided due to delayed diagnosis.^[1,4,8–10]^

Clinical symptoms include epiphora, swelling of the eyelid or punctum, erythema, pain, and redness. Clinical signs include thickening of the canalicular portion of the eyelid margin, punctal regurgitation of pus and concretions, a pouting erythematous punctum, yellowish discoloration of the canalicular region, and localized hyperemia.^[1,3,10]^ Medical and surgical management are options for treating primary canaliculitis; however, surgical management is considered the definitive treatment.^[1,5,11,12]^ In cases of recurrence after the initial treatment, conservative treatment should be avoided, and snip punctoplasty with canalicular curettage should be performed.^[1]^

At present, 1,2,3-snip punctoplasty and canalicular curettage may be an effective treatment modality for primary canaliculitis; however, previous studies have reported recurrence rates of 6.6% to 22%.^[2–5,8,11,13]^ Currently, there are no reports discussing the most appropriate surgical approach to treat recurrent primary canaliculitis; therefore, a more definitive treatment method should be considered to reduce the recurrence rate.

In the present case, a patient diagnosed with primary canaliculitis was treated using 1-snip punctoplasty and canalicular curettage; however, symptom recurrence was observed during the postoperative outpatient follow-up. Subsequent treatment with 4-snip punctoplasty and canalicular curettage successfully cured the recurrent primary canaliculitis. Accordingly, we recommend 4-snip punctoplasty and canalicular curettage as an effective treatment method for complete curettage when managing recurrent primary canaliculitis after initial treatment.

## 2 Case report

A 53-year-old female patient was admitted to our hospital with chief complaints of epiphora, discharge, eyelid flare up, and swelling near the inferior lacrimal punctum in the left eye, all of which had developed 6 months earlier. Based on the aforementioned symptoms, the patient was initially diagnosed with bacterial conjunctivitis at a local ophthalmologic clinic and administered antibiotic eye drops (0.5% levofloxacin, 4 times daily) for 6 months. However, her symptoms did not improve, and they had worsened 2 weeks prior to her admission. Subsequently, she was diagnosed with chronic dacryocystitis at a local ophthalmologic clinic and transferred to our hospital for recommended surgical treatment. The Institutional Review Board/Ethics Committee of Bucheon St Mary's Hospital approved this study. It was performed in accordance with the tenets of the Declaration of Helsinki. Written informed consent was obtained from the patient for publication of this case report and accompanying images.

**Figure 1:**
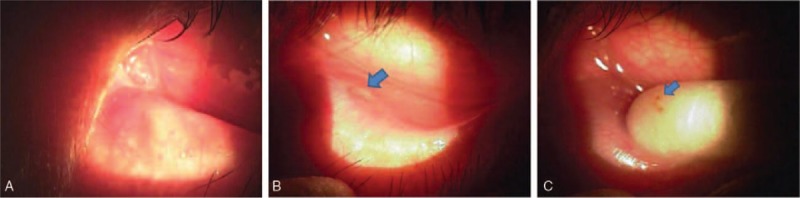
Eyelid appearance near the inferior lacrimal punctum. (A) Observation of eyelid flare up and swelling near the inferior lacrimal punctum in the left eye. (B) Observation of yellowish discharge in the lacrimal punctal orifice (arrow) with the lacrimal punctum appearing to move inward due to eyelid flare up and swelling near the inferior lacrimal punctum in the left eye. (C) Observation of concretion during punctum squeezing (arrow).

The patient had hypertension (blood pressure, 145/90 mm Hg), but no other specific underlying disease or history of previous surgery. On admission, her corrected visual acuity in both eyes was 1.0 and the intraocular pressure was normal. Slit lamp examination results showed conjunctival congestion in the inner corner of the left eye, eyelid flare up, swelling near the inferior lacrimal punctum, and yellowish discharge from the punctal orifice (Fig. 1).

There was no punctal regurgitation observed during the lacrimal sac compression test and the lacrimal irrigation test, which was performed using saline through the upper lacrimal punctum. Based on the lack of abnormal findings in the lacrimal system patency test, nasolacrimal duct obstruction, and chronic dacryocystitis could be ruled out. However, based on the yellowish discharge and concretion observed in the lacrimal punctum when the lower lacrimal punctum was squeezed using a cotton-tip applicator, a diagnosis of primary canaliculitis was made (Fig. 1).

**Figure 2:**
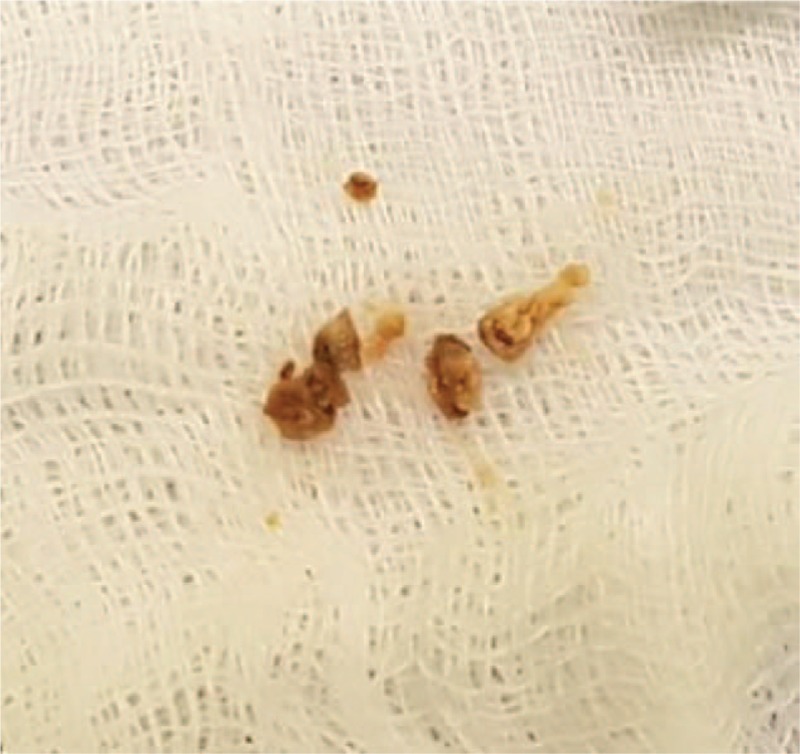
Concretions and debris removed during the 1st surgery.

Following the diagnosis of primary canaliculitis, 1-snip punctoplasty and canalicular curettage, using a 1-mm diameter chalazion curette, were performed, and lesions, such as concretions and debris, were completely removed (Fig. 2). The surgery was completed after performing the lacrimal irrigation test to verify no abnormality in the patency of the lower lacrimal system. The specimens from the lesions were sent to the laboratory for microbiologic culture and histologic examination. The microbiologic culture test could not identify the exact causative organism, but gram-positive rods were found; meanwhile, the histologic examination identified tangled clumps of filamentous organisms, which were findings consistent with a diagnosis of sulfur granules. After the surgery, the patient was prescribed oral antibiotics (cefditoren pivoxil 100 mg, 3 times daily) for 2 weeks, along with four antibiotic eye drops (0.3% gatifloxacin, 4 times daily) for 4 weeks.

**Figure 3:**
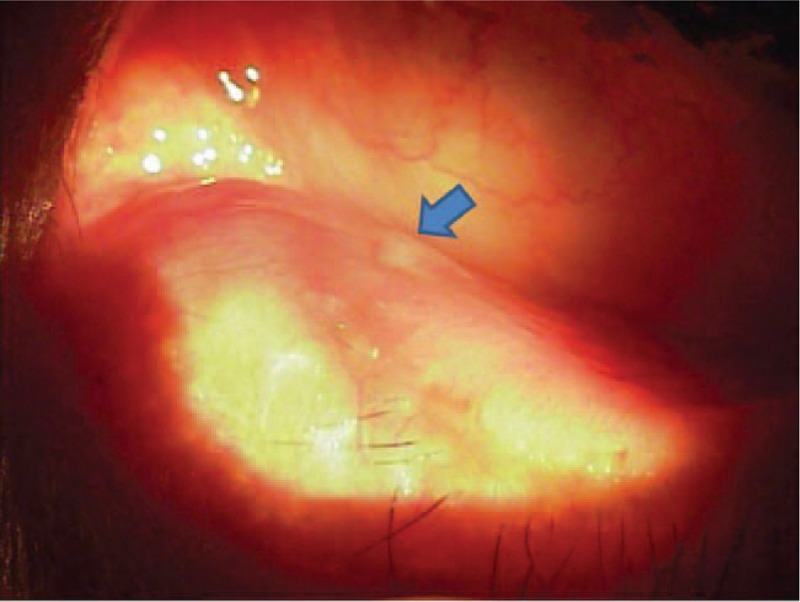
Observation of yellowish discharge from the lacrimal punctal orifice at 2 months after the surgery (arrow).

After the surgery, the patient's initial symptoms, which had caused discomfort, showed improvement, but the symptoms of epiphora and yellowish discharge from the lacrimal punctal orifice were observed during an outpatient follow-up visit 2 months after the surgery (Fig. 3).

Based on the diagnosis of recurrent primary canaliculitis, 4-snip punctoplasty and canalicular curettage were performed. Using the method described by Kim et al in a case of severe punctal stenosis,^[14]^ 4-snip punctoplasty was performed with local infiltrative anesthesia on the conjunctiva below the punctum using 2% lidocaine with 1:100,000 epinephrine. Following this, a punctal dilator was used to dilate the punctum and then the 1st vertical cut was made in a downward direction along the ampulla using Vannas scissors. Subsequently, a 2nd horizontal cut, approximately 2 mm long, was made along the roof of the canaliculus, and a 3rd vertical cut extending from the edge of the 2nd cut, was made to form the flap. Lastly, the base of the flap was removed to create a rectangular-shaped opening.

**Figure 4:**
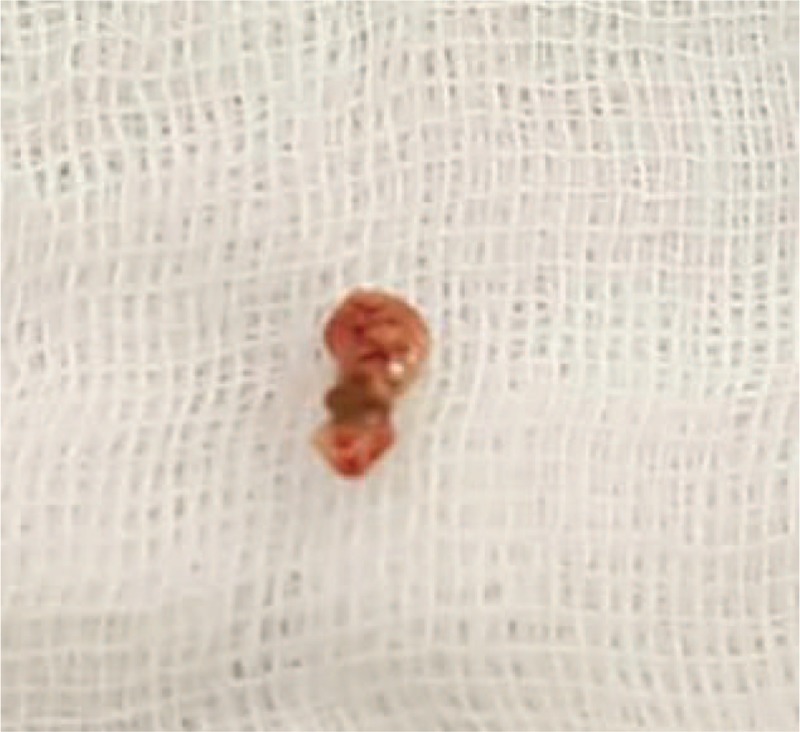
Concretions and granulomas removed during the 2nd surgery.

Next, canalicular curettage was performed using a 1-mm diameter chalazion curette, and lesions such as concretions and granuloma were completely removed. The surgery was completed by performing a lacrimal irrigation test to verify no abnormality in the patency of the lower lacrimal system. The specimens from the lesions were sent to the laboratory for microbiologic culture and histologic examination (Fig. 4).

Gram-positive rods were found; however, the microbiologic culture test could not identify the exact causative organism. Additionally, tangled clumps of filamentous organisms—findings consistent with a diagnosis of sulfur granules—were found in the histologic examination.

After the 2nd surgery, the patient was prescribed oral antibiotics (cefditoren pivoxil 100 mg, 3 times daily) for 2 weeks along with 4 weeks of antibiotic eye drops (0.3% gatifloxacin, 4 times daily).

**Figure 5:**
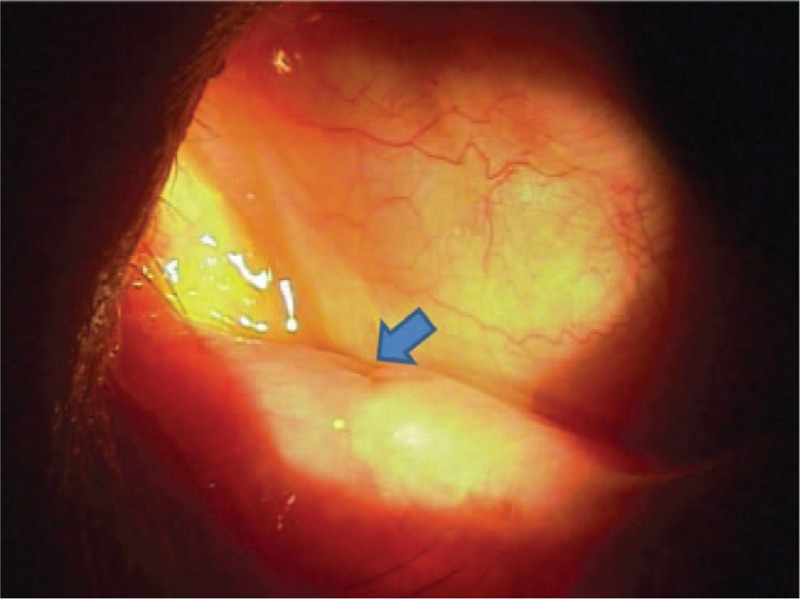
Observation of a well-formed punctum after 4-snip punctoplasty and canalicular curettage (arrow).

One month after the 2nd surgery, a well-formed punctum was observed, and all signs of epiphora, discharge, eyelid flare up, and swelling near the inferior lacrimal punctum in the left eye had disappeared (Fig. 5).

There were no findings of recurrence or complications during the subsequent 6-month follow-up period.

## 3 Discussion

Primary canaliculitis is a rare disease that involves infection in the lacrimal canaliculus. It accounts for approximately 1.4% to 2% of all lacrimal diseases; however, it is commonly misdiagnosed due to its clinical signs overlapping with other diseases that occur near the lacrimal apparatus.^[1–4,8–10]^

Literature reviews report that the rate of misdiagnosis ranges from 45% to 100%.^[5–7,9]^ As a result, there are often cases in which the appropriate treatment is not provided in timely manner due to delayed diagnosis.^[4,6–8]^ In particular, initial treatment is delayed in many cases due to symptoms of epiphora, discharge, redness, and eyelid swelling, being misdiagnosed as chronic conjunctivitis, chalazion, or chronic dacryocystitis.^[5,8,11]^ However, specific clinical signs, such as pouting of the punctum, yellowish discoloration of the canalicular region, peripunctum hyperemia, expressible punctal discharge and concretion, and negative regurgitation during the lacrimal sac compression test, can help differentiate primary canaliculitis from other diseases that present with similar symptoms.^[3,5,8–10]^

The patient in the present case was admitted for primary symptoms of epiphora, discharge, eyelid flare up, and swelling near the inferior lacrimal punctum that persisted despite long-term treatment with antibiotics for 6 months. She was ultimately diagnosed with primary canaliculitis based on gross findings of yellowish discoloration of the canalicular region, expressible punctal discharge and concretion, and negative regurgitation during the lacrimal sac compression test; therefore, the appropriate treatment was provided.

In most case reports, *Actinomyces* and *Nocardia* have been reported as the causative organisms of primary canaliculitis^[5,7–9,15–17]^; however, recent reports have revealed that *Staphylococcus* and *Streptococcus* species are the most common causative organisms.^[1,10,14,18]^ In the present case, the bacterial culture test could not identify the exact causative organism, but gram-positive rods were found. Additionally, the histologic examination identified tangled clumps of filamentous organisms, which are findings consistent with a diagnosis of sulfur granules.

Treatment options for primary canaliculitis include medical management such as warm compresses, digital massage, topical and systemic antibiotics, antifungals, corticosteroids, and hyperbaric oxygen therapy; or surgical management. Some studies have reported that medical management is somewhat effective for treating primary canaliculitis, but the frequency of recurrence may be high due to the fact that such management cannot completely eradicate the bacterial reservoir.^[5,7–9,13,18]^ In particular, it has been reported that medical treatment can have a recurrence rate of 33%^[11]^; furthermore, there have been many other reports of failed cases using medical treatment.^[2,6,7,13]^

A recent report has indicated that conservative, incision-sparing treatment had an 83.3% successful treatment rate and low recurrence rates^[19]^; however, surgical management is still considered the definitive treatment for canaliculitis.^[1,5,11,12]^

Surgical management includes punctoplasty with canalicular curettage and canaliculotomy with canalicular curettage. Further, punctoplasty and canalicular curettage can use 1-, 2-, or 3-snip punctoplasty, which has been reported to be an effective treatment for primary canaliculitis.^[3,4,10,15]^

Canaliculotomy and canalicular curettage has been reported as a safe and efficacious treatment^[7]^; however, the canaliculus plays an important role in allowing tear flow into the lacrimal sac, and canaliculotomy and canalicular curettage can ultimately cause lacrimal pump dysfunction and epiphora due to scarring or fibrosis of the canaliculus, canalicular luminal narrowing, or canalicular fistula formation. Thus, serious consideration should be given as to whether canaliculotomy should be used for treating primary canaliculitis.^[4,5,7,20]^

It has been reported that 1-snip punctoplasty and canalicular curettage is a minimally invasive and effective procedure with a high successful treatment rate of 83.3% and a low complication rate.^[4]^ In the present case, a minimally invasive surgery was planned, and 1-snip punctoplasty and canalicular curettage were performed initially. It was believed that complete canalicular curettage was achieved during the 1st surgery; however, it was found that the patient's symptoms recurred 2 months after that surgery and a 2nd surgery was planned.

It is believed that the recurrence may have been caused by incomplete removal of concretions and debris during the 1st surgery. Some concretions or debris may have been left unintentionally due to the small size of the incision.^[4]^ Therefore, widening the incision size may help reduce the likelihood of leaving concretions or debris behind, which would ultimately increase the success rate of surgery and lower the recurrence rate. In the present case, good treatment outcomes were achieved by performing 4-snip punctoplasty and canalicular curettage with punctal dilation and a wider incision.

Although 4-snip punctoplasty can be a good alternative for achieving punctal dilation and a wider incision size, it may be more invasive than 1-snip punctoplasty, which may present problems with maintaining anatomical and physiologic functioning of the lacrimal system. Another study has reported that 4-snip punctoplasty does not have a major impact on the function of the punctal sphincter or the pumping action of the canaliculus, and thus, it can be performed safely.^[4]^

**Figure 6:**
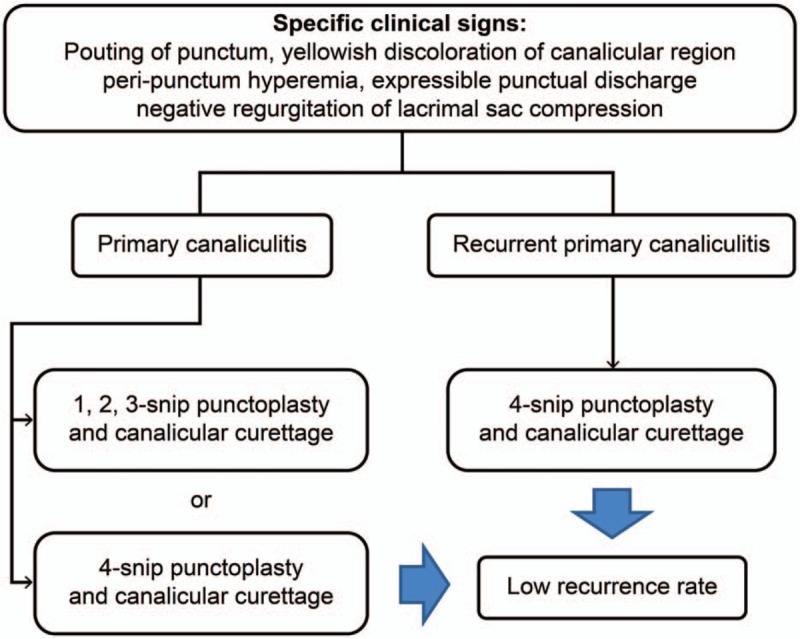
Overview of primary canaliculitis and recurrent primary canaliculitis treatment.

While 1,2,3-snip punctoplasty and canalicular curettage may be an effective treatment modality for primary canaliculitis, it is associated with a recurrence rate of 6.6% to 22%.^[2–5,8,11,13]^ Therefore, to reduce the recurrence rate, 4-snip punctoplasty and canalicular curettage, which allows a more definitive removal of canalicular concretions or debris, should be considered as the 1st-line treatment to reduce the recurrence rate, in addition to the method used for recurrent cases (Fig. 6).

We cannot conclude that 4-snip punctoplasty and canalicular curettage is the best treatment modality for recurrent primary canaliculitis based solely on the treatment outcome in the present case. This is a single case report with surgical outcomes observed over a short follow-up period. In the future, an analysis is required to investigate treatment outcomes in a larger group of patients with observations over a longer follow-up period.

Performing 4-snip punctoplasty and canalicular curettage for recurrent primary canaliculitis after 1-snip punctoplasty and canalicular curettage can increase the success rate of surgery and reduce the recurrence rates. 4-Snip punctoplasty and canalicular curettage may also be considered as the 1st-line treatment for primary canaliculitis.

## Author contributions

**Conceptualization:** Min Ho Kim, Ho Ra.

**Resources:** Min Ho Kim, Ho Ra.

**Visualization:** Min Ho Kim, Ho Ra.

**Writing – original draft:** Min Ho Kim.

**Writing – review & editing:** Min Ho Kim.
